# Profit and Loss

**Published:** 1874-10

**Authors:** Ben. H. Pratt


### THE BISTOURY.

Vol. X. ELMIRA, N. Y., OCT., 1874. No. 3.

### Contributions.

### PROFIT AND LOSS.

BEN. H. PRATT.

In the headlong endeavor of the age to reduce every species of community work to the exactness of a science; in the rash cross-cuts to advancement which theorists are continually imagining they have found, and into which they push the more practically inclined, *nolens volens;* amid the mad hootings and howlings of the visionary, who continually declaim against taking the old round-about ways our fathers traveled, and bid us accept the "new departures" and walk in the "new paths" which they in their self-constituted office of reformers have opened up; the age has well nigh lost sight of very many important facts, forgetting that rapid transit toward any objective point, be it topographical, humanitarian or educational, necessitates the neglect of the specific in the struggle after the general—the sacrifice of the physical and moral to the mental. In mental and moral as well as in natural philosophy, there is no gain without loss. Man has ceased to combat the physical axiom that time and power cannot be gained in the same piece of mechanism. He has ceased to travel by rail if his object be to study the mineral, floral or other resources of the country. Haste begets superficiality—the plodders are the truly learned. To know much of little is preferable to knowing little of much.

But aphorisms and similes are too abundant in illustration of the point we wish to get at, and the temptation to string them out is already leading us from the matter in hand.—With the above in view, therefore, let us note their application to the modern systems of education.

The hot-bed methods are now the only popular ones. The whole stress of the teacher is laid upon the one object—the stuffing of the pupil's head with what we are pleased to call learning. Bub at eight or ten years of age comes home to dinner and sets the family agog with, "I say, father, teacher says you must get me some more books; here's a note from him." Then father scans the bit of paper presented and finds that "Johnny needs Ollendorf's works on Latin, French and German—will omit Greek this term." Latin, French and German at eight! "Why, Johnny, have you completed your English? How about geography, grammar, arithmetic—have you got beyond these?" Bub laughs at his old fogy parent and informs him that he's a perfect fossil—that as for geography, it's of no account whatever in these changeful times—that his grammar days were over a year ago—can parse anything from "John's cap" up to the most difficult line in "Paradise Lost"—that his arithmetic has long been laid aside and algebraic formulæ substituted as short cuts to mathematical solutions—that all the boys of his age are scanning Latin and mouthing French and jabbering German, and he can't stand it any longer, and must have the books, so get 'em please—and so the "fossil" succumbs and wonders whether his hopeful will turn up at twenty in Congress or a lunatic asylum.

As we before stated, the whole end and aim of the latter day teacher is to cram the head at the expense of everything else. New stimulants to study have been devised—emulation has been carried to an extent that is alarming when we consider the strain upon the brain power of the child—rewards are offered—contests instituted—"show off" days are appointed—pride is appealed to—the "smart ones" are set up on an imaginary pedestal of intellectual glory that rouses the dullest youth to the exercise of the most intense brain work

—an intellectual mania takes possession of the whole school—a chronic madness to excel seizes every pupil. The strong survive it, and the prodigies, those abnormally brain endowed, live it out to the surprise of everybody.

What is the expense ?

Here is a grand mental gain, now let us see where the loss is.

It is only at the expense of the physical, moral, mental and social elements of the juvenile economy that this great gain is achieved.

As we have said, the wondrously brain endowed survive it—the rest go home to the doctor and the nurse, mentally maimed for life, and are lucky if not so affected physically as to be beyond the reach of the physician's power to reclaim or the most prudent care of the parent to overcome.

It is not the difficult thing it is frequently imagined, to incite the youthful mind to such deeds of intellectual valor as shall drown every natural physical impulse. The highly wrought pictures of the fancy that the limner Ambition paints upon the curtain that conceals the future, heightened by the glow which the glass of youthful imagination lends thereto, are so enrapturing, so alluring, so full of promise to the young, that no temptation to indulge in those youthful sports to which the world owes what of muscular strength it possesses, can compete with them. The machinations brought to bear upon bright brained youth by thoughtless or over sanguine educators, blind them to every physical need and muscular impulse. The boy that rises betimes, and possessed by the genius of ambition, incited by the hope of a glittering goal, pores the hours away in study that should be devoted to fresh lung draughts from the early morning air— who takes his breakfast hurriedly and knows no spot so enticing as his desk at school—who occupies the hours of recess and noon-spell in close application to some intricate problem in science, morals, or language—whose evenings find him first at the evening lamp and who tears himself therefrom only at the bidding of "nature's" sweet restorer, balmy sleep—such an one is sooner or later to become a victim, not so much to his own application, perhaps, as to the goads which a wily instructor is daily using to prod his ambition with, and grows up a puny creature, all intellect and no physique, without practical ballast to keep his theoretical bark from plunging and pitching in every storm of adversity that overtakes his life voyage. The modern school hero is not he who can run the fleetest, bat the strongest, pitch the swiftest, captain his nine the most successfully; but he who can conjugate the readiest, scan the closest to the rules of prosody, eliminate the unknown quantity most speedily, most accurately trace a complex demonstration, hold the position of intellectual prodigy in chief at the Academy. Physical pigmies are pigmies still, though perched on intellectual Alps.

But in the strife for the attainment of the super intellectual, what has become of the moral ? In the educator's vision, only the salient points in the pathway of learning have been noted. In his eagerness to point out the points of interest, the moral has been overlooked. Reverence for sacred things are not inculcated—respect for superior authority is not instilled—a growing tendency to regard mental acquirements as tantamount to all others—these give morality a secondary place if any place. The teacher will overlook every departure from rectitude, and almost any breach of decency in the youth who holds a high place in the intellectuality of the school room. "There is no time to do all this," says the school teacher. "If I stop in the great highway to learning to gather every other good thing that is attainable, pupilage is too short to compass more than half the way." Therefore our greatest scientists become our greaters doubters, our depreciators of virtue, our speculators in revelation, our social revolutionists, our moral pestilence breeders.

Far be it from the animus of this article to depreciate the efforts of those who are laboring for the intellectual advancement of our youth and the race. There are martyrs in the cause whose noble aims are not being given the encouragement they deserve. Improved methods of education are not to be ignored. The world grows wiser and necessarily must advance the educator's facilities with those of others. It is not these efforts that we combat, but the tendency to go to extremes. To make a specialty of brain-training to the exclusion of all other development is as wrong as it is foolish. Our preachers are becoming nought but theologians, purity of morals and sympathy with souls being sacrificed to the logic of doctrine. The head is fostered—the heart is neglected. Teachers are becoming animated cranial force pumps that see nothing to do but cerebral cramming. They stuff for a purpose, like poulterers before Thanksgiving. Our children are not being educated for that broad and noble stage whereon they are to play at men and women, but for the seven by nine platforms on which preachers and blacksmiths and lawyers and shoemakers and doctors and barbers are each to strut a bit and retire--educated narrowly, superficially, specifically, so that should one of them be deprived of his stewardship he wouldn't know to whom or what to turn for a livlihood. We believe in a more general method of education—in an education of all the powers, physical as well as mental, moral as well as social—for doctors if we may, but for wood-sawyers if we must—for preachers if we choose, for diggers if necessary.

